# Rapid improvement of *Mycobacterium kansasii* pneumonia after rifabutin, isoniazid, and ethambutol: A case report

**DOI:** 10.1002/kjm2.12608

**Published:** 2022-10-18

**Authors:** Hung‐I Kuo, Shao‐Tsung Huang, Ying‐Hsun Wu

**Affiliations:** ^1^ Division of Internal Medicine Chest Hospital, Ministry of Health and Welfare Tainan City Taiwan; ^2^ Division of Infectious Disease Chest Hospital, Ministry of Health and Welfare Tainan City Taiwan

Nontuberculous mycobacteria (NTM) species are mycobacterial species different to the *Mycobacterium tuberculosis* (TB) complex. NTM are ubiquitous in the environment and less infected immunocompetent patient.^1^ Diagnosis and further medical treatment of NTM infection generally required evidence of combination of clinical, radiological, and microbiological results. Although case reports of lung infection caused by NTM have recently increased, there is less case report described rapid improvement after adequate treatment.^2^ We herein report an immunocompetent case diagnosed *Mycobacterium kansasii* (*M. kansasii*) pneumonia that presents rapid improvement after antimycobacterial drugs used.

A 55‐year‐old male laborer with a history of smoking came to our outpatient department (OPD) in December 2021 with complaints of dyspnea and cough with productive sputum for several months. He has nephrotic syndrome under angiotensin receptor blocker follow‐up at National Cheng Kung University Hospital. Chest x‐ray (CXR) and computed tomography revealed emphysematous lung with bilateral upper bullae and large field consolidation (Figure 1A). Chronic obstructive pulmonary disease was diagnosed by pulmonary function test and inhalation therapy was commenced. We collected common sputum culture but no bacterial or fungus growth, and three samples mycobacterial sputum culture, all of which showed a positive result with acid‐fast stain, but a negative result with polymerase chain reaction for TB, and yielded *M. kansasii. M. kansasii* pneumonia was diagnosed based on the above clinical, radiological, and microbiological evidence. We started standard antimycobacterial drugs with rifampicin, isoniazid, and ethambutol first. Rifampicin was later altered to rifabutin (300 mg/day) due to intolerable fatigue and gastrointestinal upset. The patient tolerated rifabutin, isoniazid, and ethambutol well and received regular OPD follow‐ups. Follow‐up sputum culture showed no growth of *M. kansasii* after 2 months later. Follow‐up CXR showed significant improvement in bilateral upper lung consolidation 2 months later (Figure 1B). He also noted a substantial improvement in his respiratory symptoms.

**FIGURE 1:**
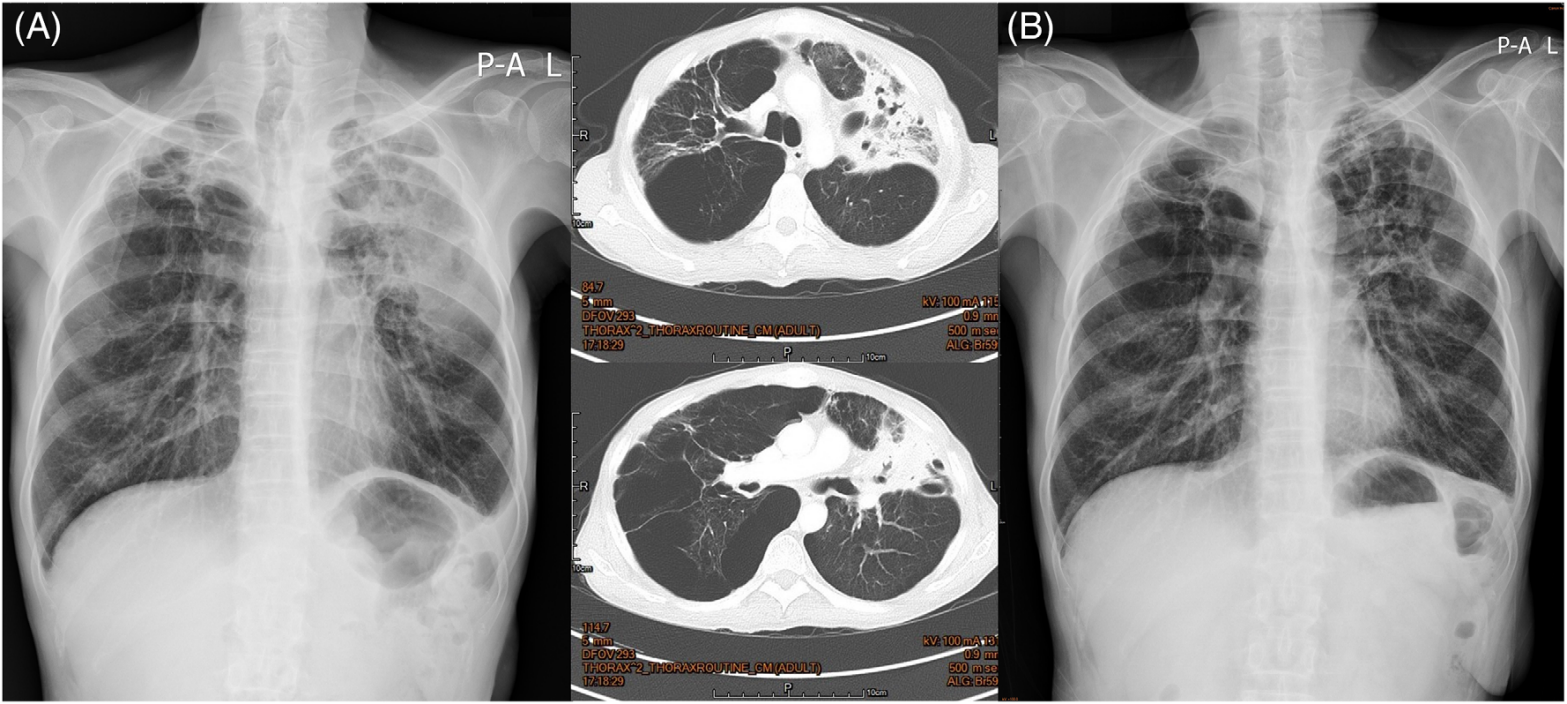
(A) Chest x‐ray (CXR) and computed tomography (CT) imagines showing an emphysematous lung with significant bullae and bilateral upper lung consolidation, which was more severe on the left side (CXR and CT taken in 2021/12). (B) CXR showing stationary emphysematous lung with significant bullae but with bilateral upper lung consolidation much improved compared to (A) (CXR taken in 2022/2).

*M. kansasii* is the second most common NTM infection after *Mycobacterium avium complex* in most countries.^1^ The clinical presentation of *M. kansasii* infection in patients described in previous case reports has varied from completely asymptomatic to extensive lung destruction with respiratory symptoms.^3^ Our case showed significant lung consolidation, similar to some severe cases reported in previous studies.^2^, ^3^ The current standard therapy for pulmonary *M. kansasii* infection is isoniazid, rifampicin, and ethambutol for 12 months after achieving sputum culture negativity.^4^ We tried standard therapy first but then switched to rifabutin due to the intolerable side effects of rifampicin. To our knowledge, no study has examined the efficacy of rifabutin against *M. kansasii* in vivo. Only a few papers have noted that rifabutin might be an appropriate alternative to rifampicin.^4^, ^5^ Our case showed significant improvement after rifabutin might prove its efficacy to against *M. kansasii* in vivo. Moreover, NTM infection generally improves slowly even under standard therapy but our patient showed outstanding improvement after only a few months of treatment.

Our case showed rapid and outstanding radiological improvement, which has rarely been reported in other studies. Thus, this case report suggests the efficacy of rifabutin against *M. kansasii* in vivo. Further study regarding alternative treatments for NTM is essential as some patients cannot tolerate the side effects associated with the current standard treatment.

## CONFLICT OF INTEREST

The authors have no conflict of interest to declare.
